# At the cutting edge: the potential of autonomous surgery and challenges faced

**DOI:** 10.1136/bmjsit-2024-000338

**Published:** 2025-03-27

**Authors:** Raghav Khanna, Nicholas Raison, Alejandro Granados Martinez, Sebastien Ourselin, Francesco Montorsi, Alberto Briganti, Prokar Dasgupta

**Affiliations:** 1 Faculty of Life Sciences and Medicine, King’s College London, London, England, UK; 2 King’s College London Faculty of Life Sciences & Medicine, London, UK; 3 King’s College Hospital NHS Foundation Trust, London, UK; 4 School of Biomedical Engineering and Imaging Sciences, King’s College London, London, UK; 5 Department of Urology, San Raffaele Hospital, Milano, Italy; 6 Department of Urology, Guy's and St Thomas’ Hospitals NHS Trust, London, London, UK

**Keywords:** Robotic Surgical Procedures, Minimally Invasive Surgical Procedures

 The past two decades have seen an exponential rise in robotic-assisted surgery (RAS). Systems such as the Da Vinci (Intuitive Surgical, USA) have catalysed a major shift from manual laparoscopy to RAS in urology, general surgery and gynaecology.^1^ Use of RAS has also increased in non-laparoscopic procedures such as biopsies, brachytherapy and hard tissue surgery. This progress and parallel advancements in Artificial Intelligence (AI) have led to interest in developing autonomous surgical robots (ASR) that will enable better precision, fewer errors and improved outcomes.^2^ This analysis provides a brief overview of progress in autonomous surgery and explores unique technical, regulatory and ethical challenges faced by ASRs.

ASRs are classified using the levels of autonomy in surgical robotics (LASR) system (figure 1).^3 4^ LASR ranges from no autonomy (level 0) to complete autonomy (level 5). Level 1 robots provide basic assistance, level 2 robots exhibit task autonomy and level 3 robots correspond to conditional autonomy. Near independent function is achieved by level 4 high autonomy robots, which operate with minimal surgeon supervision. Lee *et al* in their systematic review have provided an overview of current FDA approved ASRs.^3^ The vast majority of systems demonstrate level 1 autonomy; there are only four level 2 and three level 3 ASRs in clinical use. The review observed that there were no clinically deployed level 4 or 5 ASRs.

**Figure 1:**
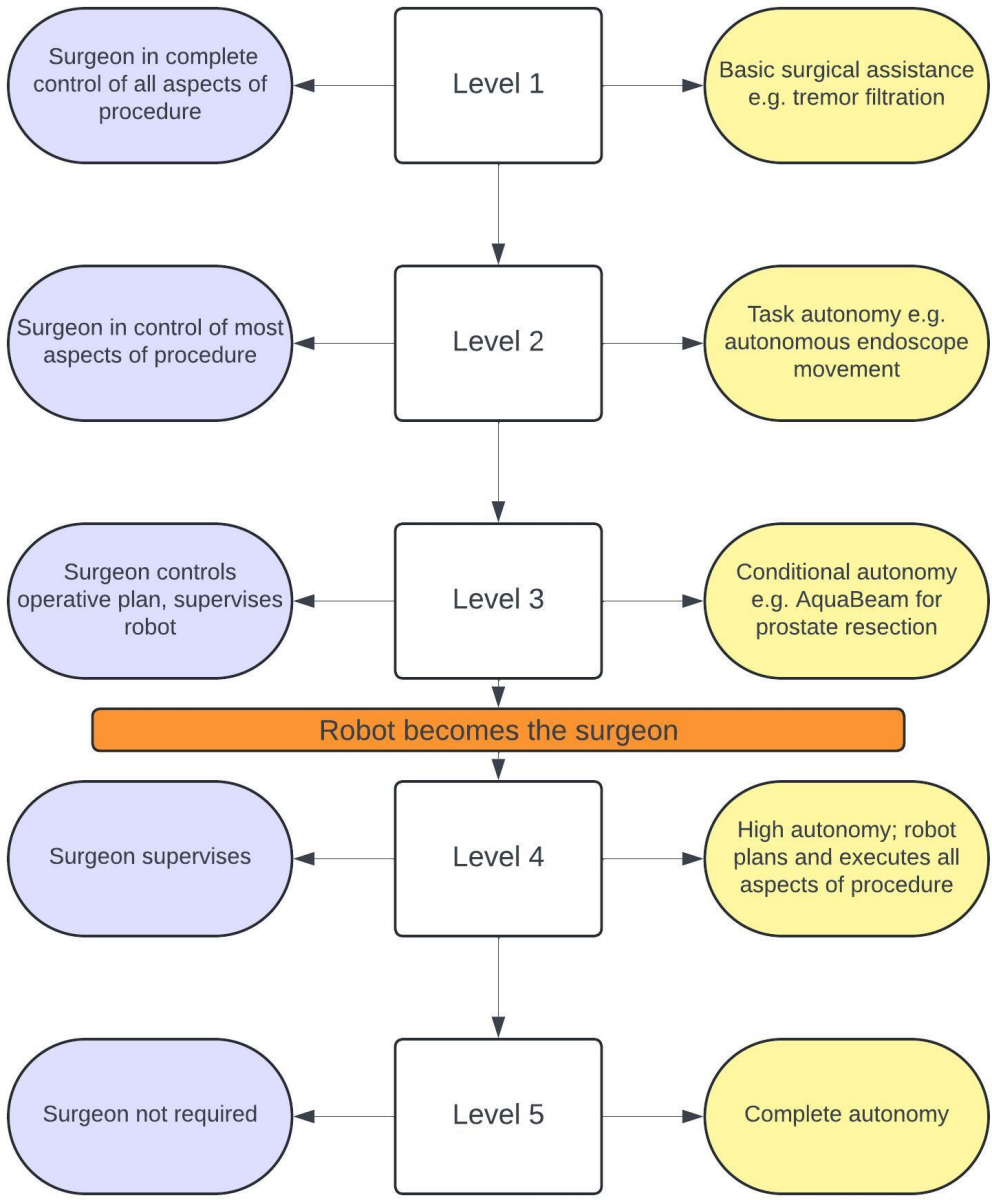
Levels of autonomy in surgical robotics (LASR). The degree of surgeon involvement (blue) decreases and robot independence (yellow) increases at higher levels.

There is a significant jump in software and hardware capabilities moving from level 2 to 3. Level 2 ASRs are ‘enhancers’, acting as force multipliers to improve surgical skill and situational awareness. For instance, the CorPath GRX robot (Siemens Healthineers, Germany) for endovascular surgery uses limited autonomous movement to aid in navigation and motion scaling.^3^ The Senhance system (Asensus Surgical, USA) for soft tissue laparoscopic surgery uses autonomous eye tracking software for endoscope movement.^3^ The autonomous component in these devices is a limited aspect of their overall robotic capability. Level 2 robots are unable to generate and execute surgical plans; instead, they enhance surgical performance.

Level 3 ASRs are ‘performers’ and carry out procedures without direct, real-time surgeon control. Robotic motion is controlled by patient-specific surgical plans based on parameters defined preoperatively by the surgeon. For example, the AquaBeam system (Procept BioRobotics, USA) for prostate resection requires a surgeon to define prostate cross section, angle and depth of ablation and resection contours.^5^ The system cannot operate outside these limits and is always supervised by a surgeon.

A detailed examination of the current landscape of FDA-approved ASRs reveals two key trends: first, most interventions are aimed at hard tissue surgery; and second, the procedures involved typically require less complex surgical manipulation compared with laparoscopic soft tissue surgery.^6^ Autonomy in soft tissue surgery is in its infancy and has focused on basic surgical skills such as suturing and tissue manipulation. An advanced prototype in this domain is the soft tissue autonomous robot (STAR), a level 3 robot for autonomous suturing.^6 7^ The latest iteration of STAR uses two robotic arms, one with a suturing tool and the other with a dual camera setup. The device employs a low-level control strategy to calculate robotic trajectories and a high-level control strategy for tissue tracking and suture path planning using a convolutional neural network (CNN). The CNN algorithm uses endoscopic feed and near-infrared markers to identify suture targets and monitor tissue displacement. Robotic movement is synchronized with breathing and a force feedback sensor allows for appropriate suture tension. The surgeon is able to select through suture plans, modify and execute them. STAR has shown promising results in performing anastomoses in phantom and in vivo porcine models, with results comparable to manual laparoscopic surgery.^6^

Apart from STAR, other groups have also explored automating tasks such as suture needle positioning, pick and place tasks, knot tying, pattern cutting and tissue manipulation through dual-arm control strategies.^2^ Research has also focused on motion and trajectory planning, object detection and collision avoidance and image and sensor-based perception technology.^2^ The EU-funded smart autonomous robotic assistant surgeon consortium has developed a two-arm robot system that can work in tandem with surgical robots such as the Da Vinci as an autonomous surgical assistant.^8^ The team has focused on the sequence of actions performed by an assistant when handing over or receiving a needle from the surgeon’s robotic arms. Kinematic and video inputs are processed by AI algorithms to predict and sequence the next robotic motion to perform and calculate velocities for the robotic arms. The system has proven itself in performing pick and place tasks, with the consortium aiming to further test it in more realistic scenarios.^8^

Achieving higher levels of autonomy (levels 4 and 5) requires the capability to act dynamically and independently. The key concept to discuss is robotic perception—the ability of a device to collect, interpret and react to information from its environment. The basic foundation of robotic perception and human perception is similar; information is gathered using sensation and interpreted using a central ‘brain’ (in the case of robotic perception, AI algorithms).

Robotic sensation can be achieved through various modalities such as vision, laser, haptic and infrared. However, adapting sensors to surgery is a significant challenge; the operational context demands miniaturized, highly accurate sensors that are resistant to sterilization, temperature changes, electrical currents and reactionary forces.^9^ In vivo sensors cannot impact the mobility of the end effector.

Even if sensation is achieved, the critical question is whether robots can mimic the human ability to think. AI has a remarkable capability to automate in structured and predictable environments.^10^ Surgery, however, is complex and unpredictable; even routine procedures will have unexpected events, new intraoperative findings that redefine operational scope, natural variations in anatomy and variations due to previous procedures. This is in contrast to other use cases of autonomy such as self-driving cars, where predictable traffic patterns, road markings and fewer variables create a less complex operating context.

Surgery demands quick and dynamic thinking from the surgeon. Polanyi’s paradox allows us to illustrate this—in simple terms, the paradox states that humans have tacit, imperceptible knowledge that cannot be replicated by AI.^10^ Surgeons are not trained through numbers; their skill develops through iterative practice and exposure to various scenarios. They acquire a unique skill set and practical knowledge that allows them to navigate new challenges in real time. The human ability to sort through a noisy and imprecise environment and make decisions is very difficult, perhaps even impossible for AI to replicate.

Moving beyond technical challenges, the safe deployment of ASRs requires appropriate oversight from regulatory bodies. Current guidelines are tailored towards lower levels of autonomy and do not account for the potential risks of increasingly independent ASRs.^3^ The majority of FDA-licensed robots in the past decade have been classified as class II (moderate risk). Robots with higher levels of autonomy should potentially be classified as class III (high risk) subject to stringent premarket evaluation.^3^ Regulators must introduce a degree of standardization by specifying universal metrics for the evaluation of ASRs, a clear pathway to clinical trials with an emphasis on patient outcomes and strategies for boosting generalizability and minimization of bias. Further guidance should be issued for monitoring of performance post deployment and protocols for reporting robot failures. Future ASRs will be connected to the wider hospital network to receive patient data from multiple sources. This is a significant challenge and ensuring interoperability between systems from different manufacturers, data communication standards, network protocols and integration into the operating room software is crucial. Regulators must address data privacy and cybersecurity concerns stemming from this.^11^ Concepts such as LASR that are basic modifications of universally used classification methods should be refined to be surgery specific.

Discussions on autonomous surgery cannot neglect the ethical aspects. Patient safety and positive outcomes must be the chief consideration when implementing new technology in healthcare. Enthusiasm around robotic surgery has previously led to the FDA issuing warnings for its unauthorized use in oncology.^12^ ASRs should be designed to solve a clinical need; they need to undergo a ‘Weizenbaum test’—to ask whether these systems are useful, rather than to implement them because the technology exists.^13^ An ASR must be developed for a specific clinical requirement; otherwise, it becomes a solution in search of a problem. Introducing autonomy to surgical robots would also require a significant augmentation to their hardware and sensor capabilities, further driving up the cost of a system that is already expensive and inaccessible to low-resource nations. Bias is also particularly important to consider; the use of unbalanced training datasets can lead to disparities in outcomes across demographics and further exacerbate health inequalities.

Accountability is another significant ethical concern. As we progress on to greater levels of autonomy, will it be the manufacturer, hospital or surgeon responsible for an error made by an ASR?^3^ Accountability further ties into the evolving role of the surgeon from performer to supervisor. Even in highly automated robotic interventions, a human operator remains in the loop for performance monitoring, oversight and support of the system.^14^ Surgeons must receive adequate training on the technical aspects of ASRs, recognizing when an error is made and appropriate ‘take-over’ procedures. Steps must also be taken to address concerns around surgeon deskilling. Protocols must be in place such that a surgical team can take over if an error or failure by an ASR necessitates a conversion to open or manual surgery.

Finally, the deployment of ASRs is dependent on patient trust. Studies have demonstrated that patients are reluctant to trust AI systems for diagnostic purposes, and this scepticism may extend to robotic surgery.^15 16^ Patients must receive accurate information about autonomous systems, including their capabilities, potential risks and the degree of operator involvement before consenting to any procedure. Surgeon’s trust is also crucial; industry should prioritize explainability, transparency and a strong evidence base. Organizations such as the United Kingdom Research & Innovation (UKRI)-funded Responsible AI UK and Trustworthy Autonomous Systems Hub are crucial to the ethical and patient-focused design of AI systems, having funded and contributed extensively to the same for the past 5 years.

Autonomous surgery is the future; the convergence of AI, robotics, sensor capabilities, material science, data integration and virtual reality will be an enabling force in the development of increasingly independent robots. Their deployment is dependent on patients and surgeons trusting these devices for effective treatment. While successes achieved in implementing autonomy to fields such as transport may create an expectation that ASRs will progress at the same pace, the challenges discussed in this paper indicate that the human surgeon will not be replaced for the foreseeable future. However, the role of the surgeon will change, and physician, patient, regulator and manufacturer input is crucial for the safe and effective use of autonomous systems.

## References

[1] Sheetz KH, Claflin J, Dimick JB (2020). Trends in the Adoption of Robotic Surgery for Common Surgical Procedures. JAMA Netw Open.

[2] Fiorini P, Goldberg KY, Liu Y (2022). Concepts and Trends n Autonomy for Robot-Assisted Surgery. Proc IEEE Inst Electr Electron Eng.

[3] Lee A, Baker TS, Bederson JB (2024). Levels of autonomy in FDA-cleared surgical robots: a systematic review. NPJ Digit Med.

[4] Connor MJ, Dasgupta P, Ahmed HU (2020). Autonomous surgery in the era of robotic urology: friend or foe of the future surgeon?. Nat Rev Urol.

[5] El-Hajj A, Nasrallah AA (2022). Step-by-step description of the Aquablation surgical treatment for benign prostatic hyperplasia. Urology Video Journal.

[6] (2022). Robot performs soft tissue surgery with minimal human help. https://www.nibib.nih.gov/news-events/newsroom/robot-performs-soft-tissue-surgery-minimal-human-help.

[7] Saeidi H, Opfermann JD, Kam M (2022). Autonomous robotic laparoscopic surgery for intestinal anastomosis. *Sci Robot*.

[8] De Rossi G, Minelli M, Roin S (2021). A First Evaluation of a Multi-Modal Learning System to Control Surgical Assistant Robots via Action Segmentation. IEEE Trans Med Robot Bionics.

[9] Heney P (2019). Challenges of building haptic feedback for surgical robots. https://www.therobotreport.com/haptic-feedback-design-challenges-surgical-robots/.

[10] Jarrahi MH, Lutz C, Newlands G (2022). Artificial intelligence, human intelligence and hybrid intelligence based on mutual augmentation. *Big Data & Society*.

[11] Alshamrani SS, Alkhudadi BA, Almtrafi SM (2022). Cyberattacks on Self-Driving Cars and Surgical and Eldercare Robots. Security and Communication Networks.

[12] (2019). FDA in brief: FDA cautions patients, providers about using robotically-assisted surgical devices for mastectomy and other cancer-related surgeries. https://www.fda.gov/news-events/fda-brief/fda-brief-fda-cautions-patients-providers-about-using-robotically-assisted-surgical-devices#:~:text=Today%2C%20the%20U.S.%20Food%20and,and%20other%20cancer%20related%20surgeries.

[13] Stilgoe J (2023). We need a Weizenbaum test for AI. Science.

[14] Fosch-Villaronga E, Khanna P, Drukarch H (2023). The Role of Humans in Surgery Automation. Int J of Soc Robotics.

[15] Richardson JP, Smith C, Curtis S (2021). Patient apprehensions about the use of artificial intelligence in healthcare. NPJ Digit Med.

[16] Torrent-Sellens J, Jiménez-Zarco AI, Saigí-Rubió F (2021). Do People Trust in Robot-Assisted Surgery? Evidence from Europe. Int J Environ Res Public Health.

